# Supplementary material for: Maternal risk factors for underweight among children under-five in a resource limited setting: A community based case control study
Source: PLoS One. 2020 May 21;15(5):e0233060. doi: 10.1371/journal.pone.0233060 (PMC7241795; doi:10.1371/journal.pone.0233060)
Supplement: S1 Appendix — (DOCX) [file pone.0233060.s001.docx]

**Questionnaire for the Survey**

**Consent Form**

Namaste! My name is ------------ (name of interviewer), from Chitwan Medical College (CMC). We are conducting is study on “**Maternal Risk Factors for underweight among under 5 children: A case-control study”** to assess the factors associated with underweight among under 5 children. The purpose of this study is to understand the maternal risk factors associated for underweight among under 5 children in Padampur VDC of Nepal. All the information will be confidential, and no any personal identifier will be disclosed anywhere in the report. There is no direct benefit from this study. However, the findings from this study will helpful to develop the strategy for addressing underweight and improve health care delivery system. The interview takes about 45-60 minutes to complete. Participation in this interview is voluntary and you can stop participating anytime without any consequences. You can also skip any questions that you wish. If you have any additional queries regarding this interview, please contact Anil Sigdel; Phone number: 9841564862.

Do you have any question regarding this telephone conversation?

Do you agree to participate in this monitoring telephone interview?

1. Yes
2. No

**Background Information**

Date: Name of Interviewer:

Ward No.: Name of Village:

1. **Child Related Information**
2. Name of Children:
3. Age of children in months
4. Weight:
5. Underweight status:
   1. Yes
   2. No
6. Sex of children:
   1. Male
   2. Female
7. Birth Order:
8. Religion
   1. Hindu
   2. Buddhist
   3. Christian
   4. Others (Specify)
9. Caste/ethnicity:
   1. Dalit
   2. Janajati/Adibasi
   3. Madeshi
   4. Muslim
   5. Brahmin/Chheteri
   6. Others
10. **Family Related Information**
11. Education of Mother
    1. Illiterate
    2. Informal Education
    3. Primary Education (Grade 1-5)
    4. Secondary Education (Grade 6-10)
    5. Higher Secondary Education (Grade 11-12)
    6. Bachelor and Above
12. Occupation of Mother
    1. Housewife
    2. Business
    3. Labor/Daily wages
    4. Services
    5. Others (Specify)
13. Income of mother:
    1. Yes
    2. No
    3. If yes, How many in a month:
14. Income of Father
    1. Yes
    2. No
    3. If yes, How many in a month:
15. How many children do you have?
16. Interval between two births (if there are more than one children):…………….. in months
17. Age of mother while giving birth to this child:
18. Who mostly cares this child:
    1. Mother herself
    2. Father
    3. Others (specify)
19. Do you smoke?
    1. Yes
    2. No
20. Do you drink?
    1. Yes
    2. No
21. **Information on Health Service Utilization**
22. Did you do ANC check-up during pregnancy?
    1. Yes
    2. No
23. If yes, how many times did you go for ANC visit?
    1. 1 time
    2. 2 times
    3. 3 times
    4. 4 or more times
24. Where did you give delivery to this child?
    1. Home
    2. Health Facility
    3. Other (Specify)
25. Who helped you during the delivery of this child? (incase delivery did not occur in HFs)
    1. Skilled Birth Attendants
    2. Relatives
    3. Others (Specify)
26. Did you go for PNC services?
    1. Yes
    2. No
27. If yes, how many times did you go for PNC checkup?
    1. 1 time
    2. 2 times
    3. 3 times
28. Did you fully immunize your children?
    1. Yes
    2. No
    3. Partially
29. If yes, did you immunize the children with following vaccines

| SN | Name of Child | BCG | DPT Hep-b | | | Polio | | | Measles | JE |
| --- | --- | --- | --- | --- | --- | --- | --- | --- | --- | --- |
|  |  |  | I | II | III | I | II | III |  |  |
|  |  |  |  |  |  |  |  |  |  |  |

1. Did you take TT vaccination during pregnancy?
   1. Yes
   2. No
2. Did you take Iron folic acid during pregnancy?
   1. Yes
   2. No
   3. Partially
3. **Environment and Sanitation Related Information**
4. Do you do anything to the water to make it safer to drink?
   1. Yes
   2. No
5. If yes, what do you usually do to make the water safer to drink?
   1. Boiling
   2. Filter
   3. Chemical/Chlorine
   4. SODIS
   5. Others (Specify)
6. What types of fuel does your household mainly use for cooking?
7. Firewood
8. Kerosene/stove
9. Biogas
10. Gas (cylinder)
11. Others (Specify)
12. What kind of stove do you have in your home? (observe it)
    1. Improved
    2. Not Improved
13. Does your Kitchen have air conditioning? (Observe it)
    1. Yes
    2. No
14. Do you have kitchen gardening?
    1. Yes
    2. No
15. Do you use insecticides/pesticides in your kitchen gardening?
    1. Yes
    2. No
    3. Sometimes
16. If yes, do you take your child with yourself during the spray of insecticides/pesticides in your kitchen gardening?
    1. Yes
    2. No
17. Do you have toilet in your home?
    1. Yes
    2. No
18. If yes, what type of toilet do you have in your home?
    1. Modern toilet
    2. Bore Hole toilet
    3. Others (Specify)
19. If No, where do you go for defecation?
    1. Forest
    2. River
    3. Field
    4. Others (Specify)
20. **Child Nutrition**
21. When did you initiated breastfeeding to your child after his/her birth?
    1. Within 1 hour
    2. After 1 hour
22. Did you feed your child with colostrum?
    1. Yes
    2. No
23. How long have you feed your child with breast milk without any supplementary food?
    1. ………………………………. Months
24. Are you still breast feeding your child?
    1. Yes
    2. No
25. If No, at what age your child stopped breastfeeding?
    1. ……………………………… Months
26. When did you have done weaning ceremony of your child?
    1. …………………………………. Months
27. How many times do your feed your child with complementary foods?
    1. 1 time
    2. 2 times
    3. 3 times
    4. 4 or more than 4 times
28. Do you feed your child with meat, fish and eggs in every week?
    1. Yes
    2. No
29. If Yes, how many times do your feed your child with meat, fish and eggs?
    1. 1 time
    2. 2 times
    3. 3 times
    4. 4 or more than 4 times
30. In the last 24 hour what are the types of foods you have given to your child?
    1. carrot, radish, potato 1 Yes 2 No
    2. Cereals, Nuts, 1 Yes 2 No
    3. Milk products (milk, curd, ghee, etc.) 1 Yes 2 No
    4. Meat, Fish 1 Yes 2 No
    5. Egg 1 Yes 2 No
    6. Fruits and vegetables rich with Vitamins 1 Yes 2 No
    7. Other fruits and vegetables 1 Yes 2 No
31. How many times do you feed your child with food when the child is sick?
    1. As usual
    2. Less than as usual
    3. More than as usual
32. **Childhood Illness**
33. What was the weight of this child during the birth?

……………………………….. Kg

1. What is the size of this child during birth?
   1. Small
   2. Normal
2. In the last 1 month did your child suffered from common cold and whooping cough?
   1. Yes
   2. No
3. If yes, how many times dis your child suffered from common cold and whooping cough?

………………………………………….times

1. In the last 1 month did your child have diarrhea?
   1. Yes
   2. No
2. If yes, how many times dis your child have diarrhea?

………………….times

1. In the last 1 month did your child have Malaria
   1. Yes
   2. No
2. In the last 1 month did your child have Measles.
   1. Yes
   2. No
3. **Household Food Security Status Related Information**
4. In the last 12 months, how frequently did you worry that your household would not have enough food?
   1. Never
   2. Rarely
   3. Sometimes
   4. Often
5. In the last 12 months, how frequently were you or any household member not able to eat the kinds of foods you preferred because of a lack of resources?
   1. Never
   2. Rarely
   3. Sometimes
   4. Often
6. In the last 12 months, how often did you or any household member have to eat a limited variety of foods due to a lack of resources?
   1. Never
   2. Rarely
   3. Sometimes
   4. Often
7. In the past 12 months, how often did you or any household member have to eat some foods that you really did not want to eat because of a lack of resources to obtain other types of foods?
   1. Never
   2. Rarely
   3. Sometimes
   4. Often
8. In the past 12 months, how often did you or any household member have to eat a smaller meal than you felt you needed because there was not enough food?
   1. Never
   2. Rarely
   3. Sometimes
   4. Often
9. In the last 12 months, how often did you or any household members eat fewer meal in a day because of lack of resources to get food?
   1. Never
   2. Rarely
   3. Sometimes
   4. Often
10. In the past 12 months, how often was there with no food t eat of any kind in your household because of lack of resources to get food?
    1. Never
    2. Rarely
    3. Sometimes
    4. Often
11. In the past 12 months, how often did you or any household member go to sleep at night hungry because there was not enough food?
    1. Never
    2. Rarely
    3. Sometimes
    4. Often
12. In the past 12 months, how often did you or any household member go a whole day and night without eating anything because there were not enough foods?
    1. Never
    2. Rarely
    3. Sometimes
    4. Often
13. **Household Assets Related Information**
14. What is the major source of income of your family?
    1. Agriculture
    2. Shops
    3. Business
    4. Service
    5. Daily wages
    6. Foreign employment
    7. Others (Specify)
15. In an average, how much saving you do in a month from different income sources?
    1. Agriculture…..
    2. Shops……
    3. Business……
    4. Services…….
    5. Daily wages……
    6. Foreign employment…..
    7. Others…….
16. Does any member of this household own any agriculture land?
    1. Yes
    2. No
17. If yes, How many Bigha of agriculture land do members of this household own?
    1. ……………………… Bigya
    2. ………………………. Kattha
    3. ……………………….. Dhur
18. Does this household own any livestock, herds, other farm animals or poultry?
    1. Yes
    2. No
19. If yes, how many of the following animals does this household own?
    1. Milk cow/bulls…………….
    2. Buffalo………………
    3. Horse/donkey or mules……………
    4. Goats………………..
    5. Sheep…………….
    6. Chicken or other poultry…………..
    7. Ducks……………
    8. Pigs………………
    9. Yaks………………
20. Does any member of this household own?
    1. A Bicycle/Rickshaw 1. Yes 2. No
    2. A Motorcycle 1. Yes 2. No
    3. An animal-drawn cart 1. Yes 2. No
    4. A car/truck/tractor 1. Yes 2. No
    5. A three-wheel tempo 1. Yes 2. No
21. Do your household have
    1. A radio 1. Yes 2. No
    2. A television 1. Yes 2. No
    3. A non-mobile telephone 1. Yes 2. No
    4. A computer 1. Yes 2. No
    5. A refrigerator 1. Yes 2. No
    6. A table 1. Yes 2. No
    7. A chair 1. Yes 2. No
    8. A bed 1. Yes 2. No
    9. A sofa 1. Yes 2. No
    10. A cupboard 1. Yes 2. No
    11. A clock 1. Yes 2. No
    12. A fan 1. Yes 2. No
    13. A invertor 1. Yes 2. No
    14. A dhiki/Jantho 1. Yes 2. No
22. Do your household have
    1. Electricity 1. Yes 2. No
    2. Drinking water 1. Yes 2. No
    3. Access to Roads 1. Yes 2. No
    4. Cable 1. Yes 2. No
    5. Internet 1. Yes 2. No
    6. Others 1. Yes 2. No
23. Observe the main material of the roof of the dwelling
    1. Natural roofing 1. Yes 2. No
    2. Rudimentary roofing 1. Yes 2. No
    3. Finished roofing 1. Yes 2. No
24. Observe the main material of the floor of the dwelling
    1. Earth/Sand 1. Yes 2. No
    2. Ceramic tiles 1. Yes 2. No
    3. Wood planks 1. Yes 2. No
    4. Cements 1. Yes 2. No
    5. Others Specify 1. Yes 2. No
25. How many rooms are there in this household?
    1. ……………………………… rooms
26. Do you have separate room which is used as a kitchen?
    1. Yes
    2. No
27. Observe the house cross ventilation
    1. Yes
    2. No

**Thank You for your support and cooperation and time for the survey.**

**QUESTIONNAIRE**

**lrtjg d]l8sn sn]h**

**s}nfzgu/ - !# , e/tk'/**

kmf/d gDa/ ===========

gd:sf/ d ====================xF' . d clxn] hg:jf:Yo ;+sfo cGtu{t lrtjg d]l8sn sn]h k|f=ln=df :gfts]fQ/ txdf cWoog/t 5' . oxL l;nl;nfdf “Maternal Risk Factors for underweight among under 5 children: A community based case control study in Padampur VDC, Chitwan” ;DaGwL zf]wkq tof/ ug{sf nflu of] cWoog ub{} 5' . ;f] cWoogsf] l;nl;nfdf tkfO{n] lbPsf ;"rgfx? Uff]Ko /flvg] 5g\ / cWoog p¢]Zosf nflu dfq k|of]u ul/g]5g\ . olb tkfO{ o; cWoogdf cfˆgf] wf/0ff lbgsf] nfuL d~h'/ x'g'x'G5 eg] d oxfFx?;Fu cg'dlt lng rfxG5' .

!= d~h'/ 5'

@ d~h'/ 5}g

***k|ZgfjnL***

ldlt M cGt/jftf{ stf{ M j8f÷uf=lj=;= g+=

ufpF÷6f]n M lhNNff M

**s_ aRrf ;DaGwL ljj/0f**

!_ gfd M @_ pd]/ -dlxgfdf _ M #_ tf}n M

Underweight **ePsf] - _** Underweight **gePsf] - _**

$_ lnª\u M ! –dlxnf @–k'?if %_ jy{ c8{/ M

^_ wd{ M ! lxGb' @–af}l4i6 #–lqmlZrog $–cGo

&_ hft M

**v_ kfl/jfl/s ljj/0f M**

!_ cfdfsf] lzIff M ========

!_ lg/If/ @_ cgf}krfl/s lzIff #_ k|fylds lzIff

$_ dfWolds %_ ±@ ^_ pRr lzIff

@_ cfdfsf] k]zf M

!_ u[x0fL @_ Jofkf/ #_ dhb'/L $_ gf]s/L %_ cGo -pNn]v ug{]_========

#_ cfdfsf] cfDbfgL M

!_ 5 @_ 5}g #=!_ olb 5 eg] slt < dlxgfdf -?k}ofFdf_=================

$_ a'afsf] cfDbfgL M

!_ 5 @_5}g $=!_ olb 5 eg] slt < dlxgfdf -?k}ofFdf_=================

%_ cfdfsf] prfO{ M ============ ^_ cfdfsf] tf}n M =================

&_ tkfO{+sf hDdf sltj6f aRrf 5g\ < M ==============================

*_ aRrfsf] hGdfGt/ -! eGbf a9L aRrf ePdf_ ==================== dlxgf

*_ of] aRrf hGdfpFbf cfdfsf] pd]/ slt lyof] < ========================================jif{df

(_ of] aRrfnfO{ w]/}h;f] s;n] x]/rfx u5{ <

!_ cfdf @_ afa' #_ cGo -v'nfpg]_ =====

!) s] tkfO{Fn] w'd|kfg ug'{x'G5 < !_ u5'{ @ _ klxn] uy{] #_ slxNo} u/]sf] 5}g

!!_ s] tkfO{Fn] dWokfg ug'{x'G5 < !_ u5'{ @ _ klxn] uy{] #_ slxNo} u/]sf] 5}g

**:jf:Yo ;]jfsf] pkof]u ;DaGwL k|ZgfjnL**

!@_ tkfO{n] of] aRrf k]6df x'Fbf ue{hfFr u/fpg' eof] < s_ u/fP v_ u/Og

!@=!_ olb u/fPsf] eP, !_ ! k6s @_ @ k6s #_ # k6s $_ $ jf ;f]eGbf a9L

!#_ of] aRrf hGdfpFbf sxfF hGdfpg' ePsf] lyof] <

!_ 3/df @_ :jf:Yo ;+:yfdf #_ cGo -v'nfpg'xf];\_ ========

!#=!_ tkfO{+n] of] aRrf hGdfpFbf s;n] ;xof]u u/]sf] lyof] < -:jf:Yo ;+:yf eGbf aflx/ ePdf_

!_ tflnd k|fKt :jf:YosdL{ @_ cfkmGt #_ cGo -v'nfpg'xf];\_ ========

!$_ of] aRrf hGldPkl5 ;'Ts]/L hfFr u/fpg'eof] < !_ u/fP @_ u/Og

!$=! olb u/fPsf] eP, !_ ! k6s @_ @ k6s #_ # k6s

!%_ tkfO{+n] of] aRrfnfO{ ;a} vf]k nufpg' eof] <

!_ nufP @_ nufOg #_ slxn]sflxF

!%=!_ olb nufPsf] eP

| qm=;+= | aRrfsf] pd]/ | BCG | DPT Hep-b | | | Polio | | | Measles | JE |
| --- | --- | --- | --- | --- | --- | --- | --- | --- | --- | --- |
| ! |  |  | I | II | III | I | II | III |  |  |
| @ |  |  |  |  |  |  |  |  |  |  |

!^_ tkfO{+n] TT vf]k nufpg' ePsf] lyof] < !_ lyPF @_ lyOg

!&_ tkfO{+n] cfO/grSsL ;]jg ug{] u'g{ePsf] lyof] <

!_ lyPF @_ lyOg #_ slxn]sflxF===============-dlxgf_

**jftfj/0fLo ;/;kmfO ;DaGwL**

!*_ s] tkfO{+ kfgL z'l4s/0f u/]/ lkpg] ug'{x'G5 <

!_ u5'{ @_ ulb{g

!*=!_ olb z'l4s/0f ug'{x'G5 eg] s'g ljlw k|of]u ug'{x'G5 <

!_ pdfNg] @_ lkmN6/ ug{] #_ Snf]l/g÷s]ldsn k|of]u

$_ 3fddf ;'sfpg] $_ cGo -v'nfpg'xf];\ _==================

!(_ vfgf ksfpgsf nflu s'g OGwgsf] k|of]u ub{} cfpg' ePsf] 5 <

!_ bfp/f @_ dl§t]n -:6f]e_

#_ uf]j/UofF; $_ UofF; -l;n]G8/_ %_ cGo pNn]v ug{]==============

@)_ tkfO{sf] 3/df s'g k|sf/sf] rNxf] 5 < - cjnf]sg ug{]_

!_ ;'wfl/Psf] @_ g;'wfl/Psf]

@!_ efG;faf6 w'jf aflx/ hfg] 7fpF 5 < -cjnf]sg ug{]_

!_ 5 @_ 5}g

@@_ tkfO{Fsf] 3/df s/];faf/L 5 <

!_ 5 @_ 5}g

@#_ tkfO{F s/];faf/L÷v]taf/Ldf ls6gfzs cf}ifwL k|of]u ug'{x'G5 <

!_ u5'{ @_ ulb{g #_ slxn]sflxF dfq

@$_ olb k|of]u ug'{x'G5 eg], s] tkfO{ s/];faf/L÷v]taf/Ldf cf}ifwL 5bf{ aRrfnfO{ ;fydf n}hfg'x'G5 <

!_ n}hfG5' @_ n}hfGg

@%_ tkO{Fsf] 3/df s'g k|sf/sf] rkL{ 5 <

!_ 5 @_ 5}g

@%=! olb 5 eg] M !_ cfw'lgs rlk{ @_ vfN8] rlk{ #_ cGo - v'nfpg'xf];\ _===================

@%=@ olb 5}g eg] M lbzf sxf ug'{x'G5 < !_ h+un @_ gbL

#_ v]taf/L tyf t/sf/L af/L $_ cGo -v'nfpg]_===

**aRrfsf] vfglkg ;DaGwL**

@^_ tkfO{Fn] of] aRrfnfO{ hGd]sf] slt ;do kl5 cfkm\gf] b'w v'jfpg' eof] <

!_ ! 306f leqdf @_ ! 306f kl5

@&_ tkfO{Fn] of] aRrfnfO{ ljuf}tL b'w v'jfpg' eof] <

!_ v'jfP @_ v'jfOg

@*_ tkfo{n] of] aRrfnfO{ slxn];Dd cfkm\gf] b'w dfq v'jfpg' eof] <

pQ/ ====================================== dlxgf

@(_ tkO{Fn] clxn] of] aRrfnfO{ b'w v'jfO/fVg' ePsf] 5 <

!_ 5' @_ 5}g

#)_ gv'jfPsf] eP aRrf slt pd]/sf] x'Fbf v'jfpg 5f8\g' ePsf] lyof] <

pQ/ M ============================================== dlxgf

#!_ aRrfsf] eftVjfO{ -kf:gL_ slxn] ug'{ eof] <

pQ/ M ================================================dlxgf

#@_ aRrfnfO{ cfkm\gf] b'w afx]s lbgsf] slt k6s cGo vfgf v'jfpg' x'G5 <

!_ ! k6s @_ @ k6s #_ # k6s $_ $ jf ;f] eGbf a9L

##_ of] aRrfnfO{ x/]s xKtf df5f, df;', c08f cflb v'jfpg] ug'{ ePsf] 5 < !_ v'jfpF5' @_ v'jfpFlbg

##=! olb v'jfpg'x'G5 eg] M !_ ! k6s @_ @ k6s #_ # k6s $_ $ jf ;f] eGbfa9L

#$_ laut @$ 306fdf tkfO{Fn] of] aRrfnfO{ s] s] vfg]s'/f v'jfpg' eof] <

pNn]v ug'{xf];\=========================================================================================

!_ cGg, ufh/, d'nf, cfn', lu7f, Eofu'/ @_ u]8fu'8L, abfd, cf]v/ cflb

#_ b'Uw kbfy{ -b'w, blx, l3pm cfbL_ $_ df5f df;'

%_ c08f ^_ le6fldg o'Qm kmnkm'nx? / t/sf/Lx?

&_ cGo kmnkm"n tyf t/sf/Lx?

#%_ aRrf la/fdL x'Fbf slt k6s vfgf v'jfpg'x'G5 <

!_ ;fljs h:t} @_ ;fljs eGbf sd #_ ;fljs eGbf a9L

**afn/f]ux? ;DaGwL**

#^_ of] aRrf hGdLbf slt lsnf] lyof] <

pQ/ M ===================================================== s]=lh=

#^=!_ of] aRrf hlGdbf sqf] lyof] < !_ ;fgf] lyof] @_ l7s} jf 7'nf]] lyof]

#&_ ljut ! dlxgfdf aRrfnfO{ ?vfvf]ls nfu]sf] lyof] < !_ lyof] @_ lyPg

#&=! olb lyof] eg] M================================================== k6s

#*_ ljut ! dlxgfdf aRrfnfO{ kvfnf nfu]sf] lyof] M !_ lyof] @_ lyPg

#*=!_ olb lyof] eg] M ===========================================k6s

#(_ ljut ! dlxgfdf aRrfnfO{ dn]l/of nfu]sf] lyof] M !_ lyof] @_ lyPg

$)_ ljut ! dlxgfdf aRrfnfO{ bf?jf nfu]sf] lyof] M !_ lyof] @_ lyPg

**3/kl/jf/sf] vfBfGg ;'/Iff cj:yfsf] ljj/0f**

$!_ laut !@ dlxgfdf tkfO{nfO{ slt k6s vfg]s'/f ck'u xf]nf eg]/ slt lrGtf nfUof]<

!= slxNo} klg nfu]g @= slxn]sflx

#= la/n}÷ w]/} yf]/} #= ;w}h;f]

$@= laut !@ dlxgfdf tkfO{ jf tkfO{sf] kl/jf/n] OR5ofOPsf] vfg]s'/f >f]tsf] cefjsf] sf/0f slt k6s vfg kfpg' ePg <

!= ;w}h;f] kfO{of] @= la/n}÷ w]/} yf]/} k6s kfO{Pg

#= slxn]sflx kfO{Pg $= slxNo} klg kfO{Pg

$#_ laut !@ dlxgfdf tkfO{ jf tkfO{sf] kl/jf/n] >f]tsf] cefjsf] sf/0f slt k6s l;ldt k|sf/sf] vfg vfg'k¥of] <

!= slxNo} klg vfg' k/]g @= slxn]sflx vfg'k¥of]

#= la/n}÷ w]/} yf]/} k6s vfg'k¥of] $= ;w}h;f] vfg'k¥of]

$$_ laut !@ dlxgfdf vfg]s'/fsf] cefjsf sf/0f tkfO{ jf tkfO{sf] kl/jf/n] cfkm'nfO{ rflxPsf] eGbf sd vfgf slt k6s vfg'k¥of] <

!= slxNo} klg vfg' k/]g @= slxn]sflx vfg'k¥of]

#= la/n}÷ w]/} yf]/} k6s vfg'k¥of] $= ;w}h;f] vfg'k¥of]

$%_ laut !@ dlxgfdf slt k6s >f]tsf] cefjsf sf/0f tkfO{ jf tkfO{sf] kl/jf/n] lbgdf yf]/} k6s vfg'k¥of] <

!= slxNo} klg vfg' k/]g @= slxn]sflx vfg'k¥of]

#= la/n}÷ w]/} yf]/} k6s vfg'k¥of] $= ;w}h;f] vfg'k¥of]

$^_ laut !@ dlxgfdf tkfO{ jf tkfO{sf] kl/jf/n] >f]tsf] cefjsf sf/0f 3/df vfg]s'/f g} gePsf] cj:yf slt k6s cfof] <

!= slxNo} klg cfPg @= slxn]sflx cfof]

#= la/n}÷ w]/} yf]/} k6s cfof] $= ;w}h;f] cfof]

$&_ laut !@ dlxgfdf tkfO{ jf tkfO{sf] kl/jf/n] vfg]s'/fsf] cefjsf sf/0f /ftfLsf] vfgf g} gvfP/ /ftL ef]s} ;'Tg' kg{] cj:yf slt k6s cfof] <

!= slxNo} klg cfPg @= slxn]sflx cfof]

#= la/n}÷ w]/} yf]/} k6s cfof] $= ;w}h;f] cfof]

**kl/jf/sf] cfly{s cj:Yfsf] ljj/0f**

$*=tkfO{sf] kl/jf/sf] d'Vo cfDbfgLsf] >f]t s] xf] <

!= s[lif %= Hofnfbf/L

@=;fgf] k;n ^= j}b]lzs /f]huf/

#= Jofkf/ -v'nfpg]_ ========== &= cGo -pNn]v ug{'xf];\_==========

$= hflu/

$(= ljleGg cfDbfgLsf] >f]tx?af6 cf};tdf dfl;s slt k};f hDdf x'G5 -?k}ofdf_

!= s[lif ==================== %= Hofnfbf/L ===================

@=;fgf] k;n============== ^= j}b]lzs /f]huf/ ============

#= Jofkf/ -v'nfpg]_ ========== &= cGo -pNn]v ug{'xf];\_==========

$= hflu/ =======================

%)= s] tkfO{sf] v]tLof]Uo hUuf÷hdLg 5 <

!= 5 @= 5}g

%)=! olb 5 eg] slt hUuf÷hdLg 5<

ljuf ================================s7\7f ===========================w'/================================

%!= tkfO{x?n] s'g} lsl;dsf] kz'÷k+IfL kfng klg ug'{ ePsf] 5 <

!= 5 @= 5}g

%!=! olb 5 eg] tnsf dWo] s'g s'g kz'÷k+IfL] 5g\ <

!= e}+;L========================= % afv|f, v;L================

@= ufO{÷uf]? ================ ^= ;'Fu'/, aF'u'/================

#= s'v'/f================ ====== &= cGo========================

$= xfF; ======================

%@= tkfO{sf] kl/jf/df oftfoftsf] s'g} ;fwgx? 5g\ < -5 jf 5}gdf uf]nf] nufpg]_

5 5}g

s=;fOsn÷l/S;f ! )

v= df]6/;fOsn ! )

u= uf8f ! )

3=6]Dkf] ! ) ª= uf8L÷6«s÷6«ofS6/ ! )

%#= tkfO{sf] 3/df ePsf 3/fo;L ;fdfgx? atfO{lbg ;Sg' x'G5 < -5 jf 5}gdf uf]nf] nufpg]_

5 5}g

s= /]l8of] ! )

v= 6]lnlehg ! )

u= df]afOn÷kmf]g ! )

3= /]lkm|lh/]6/÷lkm|h ! )

ª= knª ! )

r= 6]jn ! )

5= s';L{ ! )

h=;f]kmf ! )

em=sDKo'6/ ! )

`=cGo ! )

%$= tkfO{sf] kl/jf/df÷3/df tn pNn]lvt s] s] ;'lawfx? 5g<-atfO{ lbg' xf]nf_

!= lah'nL @= vfg]kfgL

#= af6f]÷lgsf; $= 6]lnkmf]g

%= s]a'n ^= cGo
%%= 3/÷3/sf] 5fgfsf] k|sf/ -cjnf]sg u/]/ n]Vg]_

!= sRrf

@= sRrf kSsf

#= kSsf

%^= 3/sf] e'Osf] agfj6sf]]] k|sf/ -cjnf]sg u/]/ n]Vg]_

!= df6f] $= l;d]G6

@= 6fon %= cGo -pNn]v ug{'xf];\_==================

#= sf7

%&= tkfO{sf] 3/df hDdf sltj6f sf]7fx? 5g\ < =================================== sf]7f

%*= 3/df Emofn sf] Joj:yf 5 <-cjnf]sg u/]/ n]Vg]_ != 5 @= 5}g

%(= s] 3/df 5'§} efG;fsf]7f 5 <-cjnf]sg u/]/ n]Vg]_ != 5 @= 5}g

***;f]lwPsf k|Zgsf] pQ/ lbP/ ;xof]u ug'{ePsf]df wGojfb***

***;dfKt***
