# Supplementary material for: Maternal risk factors for underweight among children under-five in a resource limited setting: A community based case control study
Source: PLoS One. 2020 May 21;15(5):e0233060. doi: 10.1371/journal.pone.0233060 (PMC7241795; doi:10.1371/journal.pone.0233060)
Supplement: Attachment — Submitted filename: PONE-D-20-02615_Priyamadhaba.pdf [file pone.0233060.s004.pdf]

PLOS ONE

Maternal Risk Factors for Underweig[illegible] among Under-five Children in a Resource Limited Setting: A Comm[illegible]nity Based Case Control Study

--Manuscript Draft--

**Manuscript Number:**	PONE-D-20-02615
**Article Type:**	Research Article
**Full Title:**	Maternal Risk Factors for Underweight among Under-five Children in a Resource Limited Setting: A Community Based Case Control Study
**Short Title:**	Maternal Risk Factors and Underweight among Children
**Corresponding Author:**	Anil Sigdel, BPH, MPH, MA(Sociology)<br>Chitwan Medical College<br>Chitwan, NEPAL
**Keywords:**	Case control Study, Risk Factors, Underweight
**Abstract:**	Previous studies conducted in Nepal have not identified the potential maternal risk for underweight among children under-five years of age in resource-poor settings. Therefore, to identify these risk factors for being underweight among children under-five years old, a community-based case-control study was conducted in a rural village in the Chitwan District in Nepal. Cases were defined as children who were diagnosed as underweight based on low weight per age, whereas controls were the children with normal weight for their age. Mothers of 93 cases and 186 controls were invited for an interview to collect the data. More than half of underweight children were female (51.6%) and nearly one third of them (31.2%) were aged 13-24 months. Nearly, 30% of the cases belonged to families in the lowest wealth quintile and 82% of cases were from food insecure families. Logistic regression analysis showed that children of mothers who were illiterate had 1.48 times the odds of being underweight compared to whose mothers were not illiterate (95% Confidence Interval [CI]: 1.53-3.07)). Children whose mother had not completed their postnatal care (PNC) were 3.16 more times likely to be underweight compared to children of mothers who completed PNC (95% CI: 1.24-8.03). The children who received care from other family members besides their mothers were 6.05 times more likely to be underweight (95% CI: 1.44-25.42); the children having mothers who had no income at all had 5.13 times the odds of being underweight (95% CI: 1.27-20.71) and children with diarrhea episodes within one month were 2.09 times more likely to be underweight (95% CI:1.02-4.31) compared to those children without any diarrhea episodes within one month. Women should be encouraged to take care of their children themselves, seek PNC services and take precautions to protect their children from diarrhea. Also, enabling factors such as education and improved income for women can help to reduce malnutrition among children.
**Order of Authors:**	Anil Sigdel
	Hardik Sapkota
	Subash Thapa
	Anu Bista
	Anil Rana
**Additional Information:**	
**Question**	**Response**
**Financial Disclosure**<br><br>Enter a financial disclosure statement that describes the sources of funding for the work included in this submission. Review the submission guidelines for detailed	The author(s) received no specific funding for this work.

Powered by Editorial Manager® and ProduXion Manager® from Aries Systems Corporation

requirements. View published research articles from *PLOS ONE* for specific examples.<br><br>This statement is required for submission and **will appear in the published article** if the submission is accepted. Please make sure it is accurate.<br><br>**Unfunded studies**<br>Enter: *The author(s) received no specific funding for this work.*<br><br>**Funded studies**<br>Enter a statement with the following details:<br>• Initials of the authors who received each award<br>• Grant numbers awarded to each author<br>• The full name of each funder<br>• URL of each funder website<br>• Did the sponsors or funders play any role in the study design, data collection and analysis, decision to publish, or preparation of the manuscript?<br>• **NO** - Include this sentence at the end of your statement: *The funders had no role in study design, data collection and analysis, decision to publish, or preparation of the manuscript.*<br>• **YES** - Specify the role(s) played.<br><br>* typeset	
**Competing Interests**<br><br>Use the instructions below to enter a competing interest statement for this submission. On behalf of all authors, disclose any competing interests that could be perceived to bias this work—acknowledging all financial support and any other relevant financial or non-financial competing interests.<br><br>This statement **will appear in the published article** if the submission is accepted. Please make sure it is accurate. View published research articles from *PLOS ONE* for specific examples.	The authors have declared that no competing interests exit.

**NO authors have competing interests**<br><br>Enter: *The authors have declared that no competing interests exist.*<br><br>**Authors with competing interests**<br><br>Enter competing interest details beginning with this statement:<br><br>*I have read the journal's policy and the authors of this manuscript have the following competing interests: [insert competing interests here]*<br><br>* typeset	
**Ethics Statement**<br><br>Enter an ethics statement for this submission. This statement is required if the study involved:<br><br>• Human participants<br>• Human specimens or tissue<br>• Vertebrate animals or cephalopods<br>• Vertebrate embryos or tissues<br>• Field research<br><br>Write "N/A" if the submission does not require an ethics statement.<br><br>General guidance is provided below. Consult the submission guidelines for detailed instructions. **Make sure that all information entered here is included in the Methods section of the manuscript.**	The ethical approval was obtained from Institutional Review Committee (IRC) of Chitwan Medical College (CMC). Prior to data collection, permission from the District Public Health Office (DPHO), Chitwan was obtained. Health workers and Female Health Care Volunteers (FCHVs) from selected Village Development Committee (VDC) were briefed on the objective of the study. Written informed consent was also taken from mothers prior to interview. In case of illiterate mothers, verbal consent from the responding mothers and written consent was obtained from other family members aged above 18 years.

Powered by Editorial Manager® and ProduXion Manager® from Aries Systems Corporation

**Format for specific study types**<br><br>**Human Subject Research (involving human participants and/or tissue)**<br>• Give the name of the institutional review board or ethics committee that approved the study<br>• Include the approval number and/or a statement indicating approval of this research<br>• Indicate the form of consent obtained (written/oral) or the reason that consent was not obtained (e.g. the data were analyzed anonymously)<br><br>**Animal Research (involving vertebrate animals, embryos or tissues)**<br>• Provide the name of the Institutional Animal Care and Use Committee (IACUC) or other relevant ethics board that reviewed the study protocol, and indicate whether they approved this research or granted a formal waiver of ethical approval<br>• Include an approval number if one was obtained<br>• If the study involved *non-human primates*, add *additional details* about animal welfare and steps taken to ameliorate suffering<br>• If anesthesia, euthanasia, or any kind of animal sacrifice is part of the study, include briefly which substances and/or methods were applied<br><br>**Field Research**<br><br>Include the following details if this study involves the collection of plant, animal, or other materials from a natural setting:<br>• Field permit number<br>• Name of the institution or relevant body that granted permission	
**Data Availability**<br><br>Authors are required to make all data underlying the findings described fully available, without restriction, and from the time of publication. PLOS allows rare exceptions to address legal and ethical concerns. See the PLOS Data Policy and FAQ for detailed information.	Yes - all data are fully available without restriction

A Data Availability Statement describing where the data can be found is required at submission. Your answers to this question constitute the Data Availability Statement and **will be published in the article**, if accepted.<br><br>**Important:** Stating 'data available on request from the author' is not sufficient. If your data are only available upon request, select 'No' for the first question and explain your exceptional situation in the text box.<br><br>Do the authors confirm that all data underlying the findings described in their manuscript are fully available without restriction?	
**Describe where the data may be found in full sentences. If you are copying our sample text, replace any instances of XXX with the appropriate details.**<br><br>• If the data are **held or will be held in a public repository**, include URLs, accession numbers or DOIs. If this information will only be available after acceptance, indicate this by ticking the box below. For example: *All XXX files are available from the XXX database (accession number(s) XXX, XXX.).*<br>• If the data are all contained **within the manuscript and/or Supporting Information files**, enter the following: *All relevant data are within the manuscript and its Supporting Information files.*<br>• If neither of these applies but you are able to provide **details of access elsewhere**, with or without limitations, please do so. For example:<br><br>*Data cannot be shared publicly because of [XXX]. Data are available from the XXX Institutional Data Access / Ethics Committee (contact via XXX) for researchers who meet the criteria for access to confidential data.*<br><br>*The data underlying the results presented in the study are available from (include the name of the third party*	All relevant data are within the manuscript and its supporting information files.

and contact information or URL).<br>• This text is appropriate if the data are owned by a third party and authors do not have permission to share the data.<br><br>* typeset	
Additional data availability information:	

Powered by Editorial Manager® and ProduXion Manager® from Aries Systems Corporation

Manuscript
Click here to access/download;Manuscript;Nutrition_plosone_final_manuscript

Maternal Risk Factors for Underweight among Children Under-five in a Resource Limited Setting: A Community Based Case Control Study

Anil Sigdel[1¶ *], Hardik Sapkota [1¶], Subash Thapa[2¶], Anu Bista[3&], Anil Rana [1&]

[1]Department of Public Health, Chitwan Medical College, Bharatpur Chitwan, Nepal

[2]Research Unit of General Practice, University of Southern Denmark, Odense, Denmark

[3]Youth and Comprehensive Education, Family Planning Association of Nepal, Lalitpur, Nepal

[*]Corresponding Author

sigdel.aanil@gmail.com

[¶]These authors contributed equally to this work.

[&]These authors also contributed equally to this work.

Abstract

Previous studies conducted in Nepal have not identified the potential maternal risk for underweight among children under-five years of age in resource-poor settings. Therefore, to identify these risk factors for being underweight among children under-five years old, a community-based case-control study was conducted in a rural village in the Chitwan District in Nepal. Cases were defined as children who were diagnosed as underweight based on low weight per age, whereas controls were the children with normal weight for their age. Mothers of 93 cases and 186 controls were invited for an interview to collect the data. More than half of underweight children were female (51.6%) and nearly one third of them (31.2%) were aged 13-24 months. Nearly, 30% of the cases belonged to families in the lowest wealth quintile and 82% of cases were from food insecure families. Logistic regression analysis showed that children of mothers who were illiterate had 1.48 times the odds of being underweight compared to whose mothers were not illiterate (95% Confidence Interval [CI]: 1.53-3.07)). Children whose mother had not completed their postnatal care (PNC) were 3.16 more times likely to be underweight compared to children of mothers who completed PNC (95% CI: 1.24-8.03). The children who received care from other family members besides their mothers were 6.05 times more likely to be underweight (95% CI: 1.44-25.42); the children having mothers who had no income at all had 5.13 times the odds of being underweight (95% CI: 1.27-20.71) and children with diarrhea episodes within one month were 2.09 times more likely to be underweight (95% CI:1.02-4.31) compared to those children without any diarrhea episodes within one month. Women should be encouraged to take care of their children themselves, seek PNC services and take precautions to protect their children from diarrhea. Also, enabling factors such as education and improved income for women can help to reduce malnutrition among children.

Introduction

Despite significant progress in the health status of children globally, under-nutrition among under children under-five remains an important public health problem in low-income countries. Undernutrition is the major cause of child mortality in low income countries. UNICEF estimates that malnutrition contributes to more than half of the nearly 12 million under-five deaths in developing countries each year, and 112 million children under-five years are underweight (<-2 weight-for-age z-scores) in low-income countries [1]. Malnutrition is a universal issue that no country in the world can overlook. About one-third of reproductive women are anemic, and each year about 20 million babies are born underweight [2]. Though significant steps are being taken to address malnutrition, there has been a slight decrease in being underweight since 2000, from 11.6% to 9.7% in 2016 [3]. Reduction of the prevalence of under-nutrition in under-five children is a top priority to reduce child mortality and morbidity [2]. Reduction in under-nutrition prevalence by 50 % between 1990 and 2015 was among the most important targets of the first Millennium Development Goal (MDG). Nevertheless, progress remains slow, and most international goals set for improving child nutrition and health were not met by 2000 [2].

Multiple factors related to both mother and children contribute to malnutrition [1]. Most of all, it is the result of a combination of inadequate dietary intake and infection. Especially in low income settings, malnourished children are more likely to die as a result of common childhood diseases, to have lifetime disabilities and weakened immune systems, and to have poor education outcomes [1]. Moreover, maternal health status plays a significant role in determining the overall health condition of a child. For instance, children who were not feed colostrum is a proximal cause and mothers' level of education as more distal cause could lead to underweight among the children.

The other major causes are maternal marital status, having a family size of five or above, children who were breast feed for less than 12 months, who had frequent diarrhea illness, and minimum dietary diversity [4,5].

Previous studies have highlighted several factors associated with the nutritional status of children. A study conducted in rural Nepal (Kamlaiya) shows that mother's age is another factor that affects the health of a child. Children who were born from mothers 24 years of age and over were found less likely to be under-weight than those with mothers below 24 years of age [6]. Additional factors (duration of breast feeding, family size, site of delivery, occupation of mother, and height of mothers) were also associated with the nutritional status of the child according to a study conducted in three districts of Nepal [8]. Other determinants of underweight include low birth weight; child's gender; illness, and household income [9]. These sorts of demographic characteristics are associated with underweight and are consistent in many other similar settings. A study conducted in Rwanda found that mothers above 35 years of age, with primary level or no education, and whose delivery was not assisted by a skilled service provider were more likely to have under-weight children [7]. Likewise, a study done in Ethiopia also found that age of child, illness, maternal decision-making power, maternal education, employment/occupation, and household income as independent and important interpreters of determining underweight children [10]. Despite the abundance of evidence in the field, little is known about the social and individual circumstances of women's life that affect the child's nutritional status, especially in a resource limited setting.

So far interventions to reduce underweight have mostly targeted affected children, such as growth monitoring, immunization, and control of infectious diseases. Interventions targeting women have not considered the impact that women's social and individual circumstances would have on the

nutritional status of the children. For instance, maternal decision-making power is a strong factor of underweight children so interventions to improve mothers' role in decision-making could contribute to alleviating the problem [10]. Likewise, the burden of being underweight can be significantly reduced by focusing an education intervention for the mothers on the importance of nutrition by community health workers [11]. Also, an emphasis on empowering women and improving the knowledge and practices of parents on appropriate infant and young child care practices plays a vital role in reducing childhood acute malnutrition [5]. Therefore, in this study, we aimed to identify the maternal risk factors for underweight among under 5 children in Padampur Village Development Committee (VDC) of Nepal.

Materials and methods

A case-control study was conducted in rural Padampur VDC of Chitwan District Nepal. Padampur VDC was selected purposively because of its high prevalence (i.e. 37%) of underweight [1].

Sampling and recruitment

Cases were underweight children (low weight for ag[illegible]). The criterion for underweight was: Z-value less than minus 2 standard deviations (SDs) below the median weight-for-age and the control were children not underweight, i.e. Z-value equal to or above minus 2 SDs. Cases and control were ascertained by using the weight-for-age indicator according to the World Health Organization’s (WHO) new growth standards from 2006 for boys and girls [12]. A household survey had been conducted prior to the study to identify the cases and control in the selected VDC. The youngest child in the family was involved if there was more than one eligible child in one family. The cases were then selected by using systematic random sampling and each case was matched with two controls in term of place of residence. Only those mothers who have provided written consent for

an interview and permit measuring the weight of their child were included in this study. Children with physical disability and children who were sick during data collection were not included. The sample for the study was calculated using Epi Info 7 statcalc with the following values based on a study [13]: confidence level (CI) = 95%, power (1-β) = 80%, case control ratio = 1:2, percentage of control exposed = 10.5%, odds ratio = 2.86. The sample size was 258 i.e. Cases=86 and controls= 172. Considering 10% non-response rate, the total sample for the study was 284. However, five interviews were incomplete and were not included in the analysis. Hence, there were 279 samples analyzed in the study i.e. 93 cases and 186 controls [Fig 1].

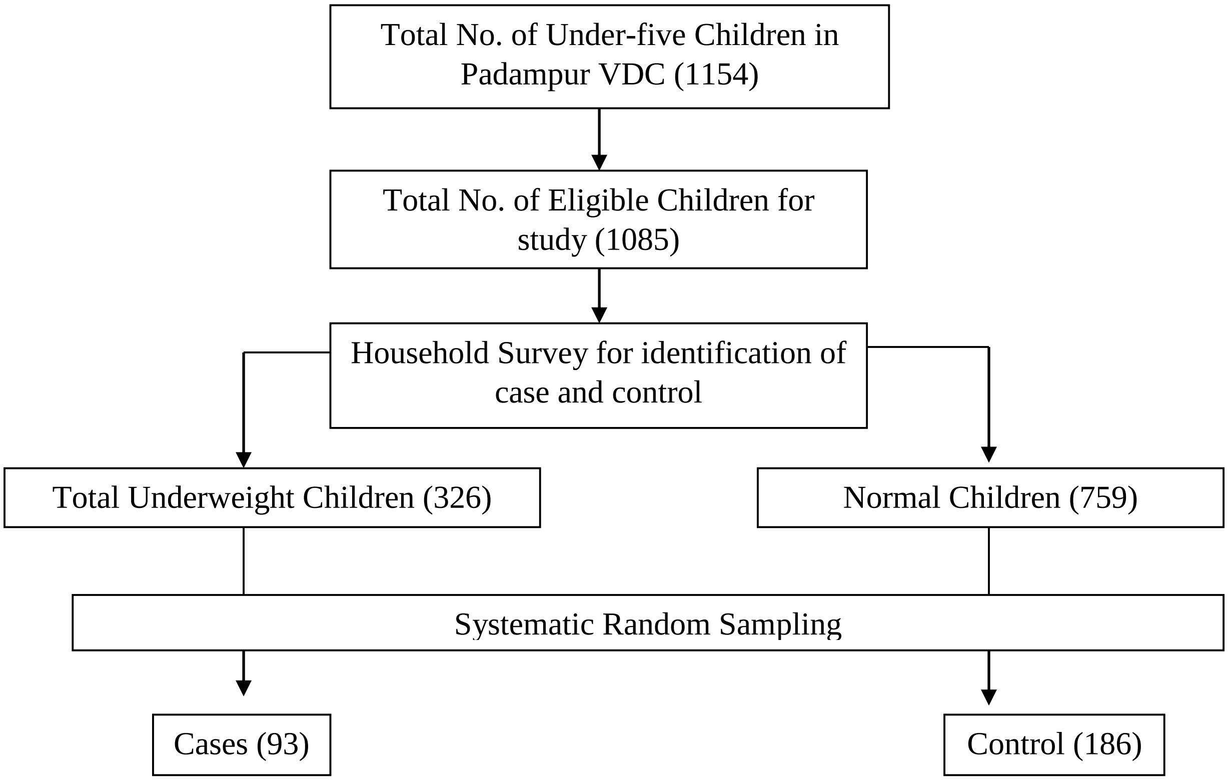

Fig 1: Sampling procedure for selection of cases and control

Questionnaire and measurement

A pre-structured questionnaire was adopted from the Nepal Health Demographic Survey (NDHS) 2011[14] and other similar previous studies conducted in Nepal [1,4,6,8,15,16]. It was then back translated into the native language (Nepali). Pre-testing of the tool was conducted in a

geographically similar VDC of Chitwan district, Jutpani to test for cultural acceptability and adaptability. A two-day training was conducted for Female Community Health Volunteers (FCHV) on interview methods, the data collection tool, ethical consideration and anthropometric measurement (i.e. weight of under-five children). Weight was measured using a calibrated Salter scale, which detects mass to the nearest half kg. The child's weight was taken without shoes and with light clothes. The weight of children less than 6 months was taken by an infant weighing scale (infant spring scale) that is used in community-based newborn care programs in Nepal [16]. Children's ages were verified either through the birth registration certificate or immunization card issued from the health facility.

Measurement of Variables

Underweight was the outcome variable of this study. Underweight was ascertained by using the weight-for-age indicator. The criterion was Z-value less than minus 2 standard deviations (SDs) below the median weight-for-age according to WHO new child growth standards 2006 [12].

Independent variables such as socio-demographic and economic factors (sex of children, ethnicity, mother's education, mother's earning status), Health service utilization factors (Antenatal Care [ANC] Visit, PNC Visit, place of delivery), Family related factors (birth order, age of mother at first child birth, number of children; height of mother; caregiver of children), history of childhood diseases (birth weight, occurrence of diarrhea, occurrence of Acute Respiratory Infection [ARI]), Feeding Practices (initiation of breast feeding, colostrum feeding, complementary feeding; exclusive breast feeding, feeding times per day, minimum food diversity, feeding during illness) were mainly reported by the child's mother during the interview. However, environmental and hygiene factors (water purification methods, availability of latrines and cooking fuel) were observed by interviewers on the day of interview. Household food security status was measured

as secure versus insecure using the household food insecurity scale developed by the Food and Nutrition Technical Assistance (FANTA) [17]. The major independent and outcome variables are shown in **Table 1**.

Table 1: Categories of variables under study

SN	Variables	Categories of Variables
**Outcome Variables**		
1	Nutritional status of under-5 children	Under-weight (low weight for age)
**Independent Variables**		
**Socio-demographic Variables**		
2	Education of Mother	Illiterate; Literate
3	Sex of child	Male; Female
	Mother's earning status	Yes; No
**Family Related Variables**		
4	Child's Birth order	Second or less; Third or more
5	Height of Mother	Less than 150 cm; 150 cm and more
6	Care giver of children	Mothers; Others
	Age of mother at first child birth	Between 20-30 Years; Others
**Health Service Utilization Factors**		
7	ANC Visit	Less than 4 visits; 4 or more visit as per protocol
8	Place of Delivery	Health Facility; Home and other place
9	PNC visit	Yes; No

**Environmental and Hygiene Related Factors**		
10	Cooking Fuel	Woods; LPG gas
11	Availability of toilet	Yes; No
12	Types of Toilet	Dug well Latrine; Modern
13	Water Purification Methods	Yes; No
**Feeding Practices Variables**		
14	Initiation of Breast Feeding	Within one hour; After one hour
15	Colostrum Feeding	Yes; No
16	Exclusive Breas feeding	6 months or above; Less than 6 months
17	Feeding times per day	Less than 3 times; 3 times or more
18	Minimum Food diversity	Standard (4 or more types of food out of 7 types (Grains, roots and tubers; Legumes and nuts; dairy products; Flesh foods; Eggs; Vitamin A rich fruits and vegetables and Other fruits and vegetables) consumed in a day; Below Standard (consumed less than 4 types of food)
19	Feeding during illness	Less than usual day; As usual or more
**History of Childhood diseases related variables**		
20	Birth weight	Less than 2.5 kg; 2.5 Kg or more
21	Occurrence of ARI	No ARI; One or more times
22	Occurrence of diarrhea	No Diarrhea; One or more times

23	**Food Security Status**	Secure; Insecure

Data collection

At first, FCHVs approached the mothers of the selected children and asked for their consent to measure the weight of their children and to participate in an interview. Face to face interviews took place in a separate room of the selected household by FCHVs using the semi-structured questionnaire. On average, it took about 45 minutes to complete one interview.

Ethical Considerations

The ethical approval was obtained from Institutional Review Committee (IRC) of Chitwan Medical College (CMC). Prior to data collection, permission from the District Public Health Office (DPHO), Chitwan was obtained. Health workers and FCHVs from the selected VDC were briefed on the objective of the study. Written informed consent was also taken from mothers prior to interview. In the case of illiterate mothers, verbal consent was obtained from the responding mothers and written consent was obtained from other family members. Also, all mothers were explained about the objectives, benefits, risks and use of the study prior to the interview. No personal identifiers were collected during the interview. An alpha-numeric code was assigned to each respondent to maintain confidentiality. After completing this procedure, the interview was conducted, and the weight of children was measured.

Data Analysis

Data was analyzed using SPSS version 22 for windows. A chi-square test was used to compare the proportions between cases and controls. Logistic regression analysis was carried out to identify factors associated with being underweight. All the variables with a p-value of 0.20 in bi-variate analysis were entered in a multi-variate model. Stepw[illegible] forward modeling was used with a p value

of 0.20 for entry and 0.10 for exit. All polychotomous variables were dichotomized as seen in **Table 1** for the logistic regression analysis. Hosmer and Lemeshow goodness of fit test was used to test the fitness of the model.

Results

Approximately one-third of cases (31.2%) were in 13–24 months of age. The mean of age of cases and controls was 22.1 and 22.4 months respectively. Half of all cases were female (51.6%). Hindu children (69.9%), Disadvantaged Janajatis (caste/ethnic minority groups) (80.6%), households with food insecurity (82.8%) and lowest quintile households (29%) accounted for a higher proportion of cases as summarized in **Table 2.**

Table 2: Socio-demographic and economic characteristics of cases and control

**Characteristics**	**Nutritional Status (weight for age)**		**Total n=279 (%)**
	**Case n=93 (%)**	**Control n=186 (%)**	
**Age of children(mon[illegible]**			
0-12	28 (30.1)	61 (32.8)	89 (31.9)
13-24	29 (31.2)	44 (23.7)	73 (26.2)
25-36	19 (20.4)	43 (23.1)	62 (22.2)
36-48	12 (12.9)	23 (12.4)	35 (12.5)
49-60	5 (5.4)	15 (8.1)	20 (7.2)
Mean age (in months)	22.1 ± 14.9	22.4 ± 15.8	22.3 ± 15.5

Characteristics	Nutritional Status (weight for age)		Total
	Case<br>n=93 (%)	Control<br>n=186 (%)	n=279 (%)
**Sex of the children**			
Male	45 (48.4)	115 (61.8)	160 (57.3)
Female	48 (51.6)	71 (38.2)	119 (42.7)
**Religion**			
Hindu	65 (69.9)	123 (66.1)	188 (67.4)
Buddhist	12 (12.9)	37 (19.9)	49 (17.6)
Christian	16 (17.2)	26 (14.0)	42 (15.1)
**Ethnicity**			
Dalit	11 (11.8)	18 (9.7)	29 (10.4)
Disadvantaged Janajatis	75 (80.6)	142 (76.3)	217 (77.8)
Relatively advantaged Janajatis	3 (3.2)	7 (3.8)	10 (3.6)
Upper caste groups	4 (4.3)	19 (10.2)	23 (8.2)
**Educational status of the mother**			
Illiterate	30 (32.3)	30 (16.1)	60 (21.5)
Informal education	12 (12.9)	19 (10.2)	31 (11.1)
Primary education	47 (50.5)	89 (47.8)	136 (48.7)
Secondary education	3 (3.2)	36 (19.4)	39 (14.0)

Characteristics	Nutritional Status (weight for age)		Total
	Case<br>n=93 (%)	Control<br>n=186 (%)	n=279 (%)
Inter-mediate education (10+2)	1 (1.1)	9 (4.8)	10 (3.6)
Higher education	0 (0.0)	3 (1.6)	3 (1.1)
**Occupation of mother**			
Housewife	87 (93.5)	152 (81.7)	239 (85.7)
Involved in income generating works	6 (6.5)	34 (18.3)	21 (7.5)
**Wealth Quintile**			
Low	27 (29.0)	28 (15.1)	55 (19.6)
Second	26 (28.0)	30 (16.1)	56 (20.1)
Middle	19 (20.4)	37 (19.9)	56 (20.1)
Fourth	12 (12.9)	44 (23.7)	56 (20.1)
Highest	9 (9.7)	47 (25.3)	56 (20.1)
**Food Security status**			
Insecure	77 (82.8)	108 (58.1)	185 (66.3)
Secure	16 (17.2)	78 (41.9)	94 (33.7)

Stepwise forward logistic regression analysis was carried out with underweight as a dependent variable and nine independent variables with a p value less than 0.20 in bivariate analysis. The final model is summarized in **Table 3**. Children of mothers who were illiterate were 1.5 times

more likely to be underweight than children of mothers who were literate (95% CI: 1.53-3.08). Children of mothers who did not received PNC services were 3.12 times more likely to be underweight than the children of mothers who visited PNC (95% CI: 1.24-8.03). Children who received care from other members of the family besides their mother were 6.04 times more likely to be underweight than control (95% CI: 1.44-25.42). Similarly, mothers who had no income were 5.13 times more likely to be underweight compared to those who had monthly income (95% CI: 1.27-20.71). Likewise, the episodes of diarrhea and underweight were found to be significantly associated. It was found that children who had one or more diarrhea episodes with in a one-month period were 2.09 times more likely to be underweight than those children who did not experience episodes of diarrhea (95% CI 1.02-4.31). Other factors like age at child birth, sex of children, ANC visit, breast feeding within one hour of delivery and exclusive breast feeding were not significantly associated with underweight in the multivariate analysis as shown in **Table 3**.

Table 3: Factors Associated with underweight among under 5 children

**Variables**	**COR (95% CI)**	**AOR (95% CI)**
**PNC Visits**		
No (Less than 3)	3.02 (1.65-5.51)	3.31 (1.40-7.78) *
Yes (3 or More)	1	1
**Care giver of children**		
Others	3.38 (1.46-7.87)	5.51 (1.48-20.54) *
Mother	1	1
**Income of mother**		

No	3.72 (1.51-9.16)	4.29 (1.25-14.78) *
Yes	1	1
**Age at Child Birth**		
<20 and >30 years	2.34 (1.41-3.90) *	1.74 (0.84-3.57)
20-30 years	1	1
**Sex of children**		
Female	1.73 (1.04-2.86) *	0.62 (0.30-1.26)
Male	1	1
**Educational Status of Mothers**		
Illiterate	2.47 (1.38-4.44) *	1.48 (1.53-3.07) *
Literate	1	1
**ANC Visit**		
No (Less than 4)	2.64 (1.47-4.73) *	1.63 (0.74-3.59)
Yes (4 or More)	1	1
**Breast feeding Start**		
After one hour	2.03 (1.22-3.37) *	1.35 (0.64-2.82)
Within one hour	1	1
**Exclusive breast feeding**		
<6 and ≥6 Months	2.02 (1.21-3.36) *	1.53 (0.71-3.30)
Up to 6 months	1	1
**Episodes of Diarrhea**		
One or more times	2.35 (1.41-3.93) *	2.09 (1.02-4.31) *
No diarrhea	1	1

*statistically significant at $p<0.05$, CI= Confidence Interval; COR= Crude Odds Ratio; AOR= Adjusted Odds Ratio; 1= Reference

Children of mothers who have not received PNC services, children who received care from others besides their mother, mothers having income and children with one or more diarrhea episodes were statistically significant independent factors for underweight among children under 5 years of age. However, other factors like age at child birth, sex of children, ANC services received by mothers, breast feeding within one hour of birth, exclusive feeding, colostrum feeding, and complementary feeding were not statistically significant risk factors associated with underweight. The p value for Hosmer and Lemeshow goodness of the fit was 0.85. The Nagelkerke R Square for the model was 0.38.

Discussion

The study conducted in Zambia concluded that children with illiterate mothers were 1.12 times more likely to be underweight (95% CI: 1.11-1.12) [18]. Another study in Ethiopia also showed that children whose mothers have a basic education were 0.18 times less likely to be underweight than illiterate mother (95% CI: 0.08-0.41) [19]. All these findings resembled with this study, where the children of illiterate mothers were 1.5 times more likely to be underweight. Previous studies have shown that educated mothers are better informed about optimal child care practices, have better practices in terms of hygiene, feeding and childcare during illness, have a greater ability to use the health system, are more empowered to make decisions and are more likely to have financial resources to care for and feed children [7].

Income status of mothers was a risk factor associated with underweight among children under-five years of age. This study showed that children whose mothers had no income were more than three

times more likely to be underweight as compared to those mothers who have monthly income. This finding is aligned with the study conducted in Kailali district of Nepal which showed that risk factors for stunting comprised mothers without earning [15]. Likewise, a study conducted in Botswana showed that children of unemployed parents were more likely to be underweight [20]. These studies both show that nutritional status of children is highly associated with the earning status of mothers. A possible explanation could be that mothers who have income have better access to the health system and are more empowered to make decisions to care for and feed children.

Likewise, children who received care from other members of the family besides their mother were more than three times more likely to be underweight than those who were cared by their mother. These findings resembled the study conducted in Botswana, which showed that children raised by other members of the household were 5.67 times more likely to be underweight than raised by their own mothers (95% CI: 1.30-24.73) [20]. This might be because mothers are more connected and are more protective towards their children than other family members. Any minor deviation in health and hygiene habits of children are easily noticed by mother and take actions timely to prevent further complications.

PNC visits were highly associated with underweight in multivariate logistic regression analysis. Children whose mothers did not visit PNC were more than three times more likely to be underweight than the children of mothers visited PNC. The finding resembled the study conducted in Kunchha VDC of Nepal where children whose mothers did not visit were more than seven times more likely to be malnourished [21]. PNC is the period after the delivery of the child which includes routine clinical examination and observation of the woman and her child. Mothers visiting

PNC could be more informed about their health and the health of their children and seek health services more frequently.

The occurrence of diarrhea within a one-month period is a risk factors associated with underweight among under five children. Children suffering from diarrhea one or more times during one-month period were two times more likely to be underweight than those who do not suffer from diarrhea. This finding aligns with the study conducted in Vietnam where it was found that children with diarrhea in the last two weeks are more than two times more likely to be underweight [8]. Likewise, other studies also revealed that child illness has a significant relationship with underweight of children [5,20]. This is clear because there is a reciprocal relationship with diarrhea leading to malnutrition while malnutrition predisposes to diarrhea. Infections play a major role in the etiology of under nutrition because they result in increased needs and high energy expenditure, lower appetite, nutrient losses due to vomiting, diarrhea, poor digestion, malabsorption of nutrients and disruption of metabolic equilibrium [10].

Conclusions

In this study, we found that children whose mother has not completed her postnatal care, and who were illiterate, the children who received care from other family members besides their mothers, the children whose mothers had no income at all, and children with diarrhea episodes within one month were found to be at a significant risk of being underweight. It is recommended that women should be encouraged to take care of their children oneself, seek PNC services and take precautions to protect children from diarrhea. Also, increasing education and income generation for women can help to reduce malnutrition among children.

Limitations

This study has some limitations. First, due to the retrospective nature of the study, it could be argued that the observed associations are due to recall bias. Second, the study was carried out in Padampur VDC of Nepal so the findings may have limited generalization. Third, the limited number of sample size may have led to a low statistical power to detect statistically significant differences. Forth, the use of self-reports might raise concerns about recall bias and social desirability tendencies. However, the strong study design and consistent results with previous studies conducted in Nepal and elsewhere suggest that the observed associations are valid.

Acknowledgements

The authors would like to thank District Public Health Office (DPHO) Chitwan, Padampur Village Development Committee (VDC) and all Female Health Care Volunteers for their help and support during the field work of this study. We would like to extend our sincere gratitude towards Natalie Linton and Hannah Marqusee for professional editing of the manuscript.

Authors Contributions

Conceptualization: Anil Sigdel

Formal Analysis: Anil Sigdel and Hardik Sapkota

Investigation: Hardik Sapkota, Anu Bista and Anil Rana

Methodology: Hardik Sapkota and Anil Sigdel

Software: Anil Sigdel and Hardik Sapkota

Writing- Original Draft Preparation: Anil Sigdel, Anu Bista, Subash Thapa and Anil Rana

Writing- Reviewing & Editing: Anil Sigdel, Subash Thapa, Anu Bista and Hardik Sapkota

References

1. Ruwali D. Nutritional Status of Children Under Five Years of Age and Factors Associated in Padampur VDC , Chitwan. Heal Prospect [Internet]. 2011;10:14–8. Available from: https://www.nepjol.info/index.php/HPROSPECT/article/view/5639

2. UNICEF, WHO, World Bank Group, United Nations. Levels and Trends in Child Mortality: Estimates Developed by the UN Inter-agency Group for Child Mortality Estimation. 2019;52.

3. El Taguri A, Betilmal I, Mahmud SM, Monem Ahmed A, Goulet O, Galan P, et al. Risk factors for stunting among under-fives in Libya. Public Health Nutr. 2009;12(8):1141–9.

4. Sah N. Determinants of child malnutrition in Nepal: a case analysis from Dhanusha, Central Terai of Nepal. J Nepal Health Res Counc [Internet]. 2004;2(2):50–4. Available from: http://www.jnhrc.com.np/index.php/jnhrc/article/viewFile/89/86

5. Miskir A, Godana W, Girma M, G/Miskel F. Determinants of Acute Malnutrition among Under-Five Children in Karat Town Public Health Facilities, Southern Ethiopia: A Case Control Study. Qual Prim Care [Internet]. 2017;25(4):242–52. Available from: http://search.ebscohost.com/login.aspx?direct=true&db=cin20&AN=125191727&site=ehost-live

6. Khatri RB, Mishra SR, Khanal V, Choulagai B. Factors associated with underweight among children of former-Kamaiyas in Nepal. Front Public Heal. 2015;3(JAN):1–6.

7. Mukabutera A, Thomson DR, Hedt-Gauthier BL, Basinga P, Nyirazinyoye L, Murray M. Risk factors associated with underweight status in children under five: an analysis of the

2010 Rwanda Demographic Health Survey (RDHS). BMC Nutr [Internet]. 2016;2(1):1–12. Available from: http://dx.doi.org/10.1186/s40795-016-0078-2

8. Dhungana GP. Nutritional Status and the Associated Factors in Under Five Years Children of Lamjung, Gorkha and Tanahun Districts of Nepal. Nepal J Stat. 2017;1:15–8.

9. Sangam S, Naveed A, Athar M, Prathyusha P, Moulika S, Lakshmi S. International Journal of Health Sciences and Research. 2015;5(1):156–64.

10. Tosheno D, Mehretie Adinew Y, Thangavel T, Bitew Workie S. Risk Factors of Underweight in Children Aged 6-59 Months in Ethiopia. J Nutr Metab. 2017;2017.

11. John J, John J. Prevalence and risk factors associated with underweight among under-five children in a rural area of Puducherry. Muller J Med Sci Res [Internet]. 2018;9(1):7. Available from: http://www.mjmsr.net/text.asp?2018/9/1/7/223903

12. Organization WH. Weight for age: Birth to 5 years (z-scores). Vol. 28, Printwear. 2015.

13. Nahar B, Ahmed T, Brown KH, Hossain MI. Risk factors associated with severe underweight among young children reporting to a diarrhoea treatment facility in Bangladesh. J Heal Popul Nutr. 2010;28(5):476–83.

14. Ministry of Health and Population (MoHP) NE and III. Nepal Health Demographic Survey 2011. 2012.

15. Paudel R, Pradhan B, Wagle RR, Pahari DP, Onta SR. Risk factors for stunting among children: A community based case control study in Nepal. Kathmandu Univ Med J. 2012;10(39):18–24.

16. Adhikari D, Khatri RB, Paudel YR, Poudyal AK. Factors Associated with Underweight

among Under-Five Children in Eastern Nepal: Community-Based Cross-sectional Study. Front Public Heal. 2017;5(December):1–9.

17. Coates J, Swindale a, Bilinsky P. Household Food Insecurity Access Scale (HFIAS) for measurement of food access: indicator guide. Washington, DC Food Nutr Tech …. 2007;(August):Version 3.

18. Nzala SH, Siziya S, Babaniyi O, Songolo P, Muula AS, Rudatsikira E. Demographic , cultural and environmental factors associated with frequency and severity of malnutrition among Zambian children less than five years of age. J Public Heal Epidemiol [Internet]. 2011;3(August):362–70. Available from: http://www.academicjournals.org/jphe

19. Demissie S. Magnitude and Factors Associated with Malnutrition in Children 6-59 Months of Age in Pastoral Community of Dollo Ado District, Somali Region, Ethiopia. Sci J Public Heal. 2013;1(4):175.

20. Kadima YE. Factors Influencing Malnutrition Among Children Under 5 Years of Age in Kweneng West District of Botswana. 2012;(Novr):76.

21. Dhungana GP. Nutritional Status of Under 5 Children and Associated Factors of Kunchha Village Development Committee. 2013;38–42.

Competing Interests

The authors have declared that no competing interest exits.

Survey Data set

Click here to access/download
Supporting Information
Final_data_nutrition.sav

Click here to access/download
Supporting Information
Nutrition_plosone_final_manuscript_hm.docx
