# Supplementary material for: Maternal risk factors for underweight among children under-five in a resource limited setting: A community based case control study
Source: PLoS One. 2020 May 21;15(5):e0233060. doi: 10.1371/journal.pone.0233060 (PMC7241795; doi:10.1371/journal.pone.0233060)
Supplement: Attachment — Submitted filename: Response to Reviewers.docx [file pone.0233060.s005.docx]

Date: 13-04-2020

Dear Reviewer,

**Submission of the revision of the Manuscript (PONE-D-20-02615)**

Thank you very much for your constructive feedback on our manuscript. We found the comments very helpful and have revised the manuscript accordingly. We hope that our revised manuscript will be considered for publication.

For your convenience, we reproduce below in full the feedback received, followed by our responses.

Thank you, we really do appreciate your and the reviewers’ time and effort. Please contact us if you need any further revisions or information.

Kind regards

Anil Sigdel

On behalf of the coauthors

| **SN** | **Reviewers Comment** | **Author Response** |
| --- | --- | --- |
| 1 | Here authors objective  to study chronic malnutrition or acute malnutrition | Thank you for the comment. In this study, our objective was to study the acute form of malnutrition using community-based data. |
| 2 | Why you took weight for age? What you think if we have taken stunting (Yes/No) ...Will be it better? I thinks authors are interested in chronic malnutrition | In the present study, we used ‘weight-for-age’ to identify the children with underweight (acute malnutrition). We really appreciate your suggestion that investigating the factors for stunting, i.e., chronic malnutrition would also very relevant in the present context, and this is what we have planned to do as a future project that will analyze the Demographic and Health Survey data from 2001 to 2016. |
| 3 | Percentage of control exposed 10.5%. Why you took this? | As it has been made clear in the manuscript that the percentage of control exposed, i.e., 10.5% was used to calculate the data using Epi Info statcalc. Please refer to the picture attached for the parameters that were used while calculating the sample size for this study. |
| 4 | What was the precision of the instrument? Near to half Kg? | The precision of the instrument was 100 grams, and this information has been mentioned in the ‘questionnaire and measurement’ sub-section of the revised manuscript. |
| 5 | How you weigh the older children? 4/5 years (who were walking). by salter (Maximum weight range for the use salter scale) | Thank you for the comment and as you have said, the weight of 4/5 years children were measured by salter scale. The maximum weight range for the use of salter scale is considered to be 25 kg and can be used up to children under 9. |
| 6 | Are the persons (FCHVs) who collected the data, were also involved in delivery of health care services (ANC, PNC). If Yes, there is possibility of interviewer bias in the study.  This could be limited with independent data collector who were not involved with health care services delivery. | Thank you for pointing this out. As the research team was also aware of the possibility of interviewer bias and this is why FCHVs, unlike other community health workers, who are not directly involved in delivering the health care services were involved to conduct the field work. FCHVs in Nepal work as health message communicators in the community, and as such, they have no direct influence on the population of interest in this study through their personal and professional relationship in relation to health services provision. |
| 7 | Forward stepwise logistic regression model ..... | The suggested change has been incorporated. |
| 8 | add SD (+-SD) in heading | The suggested change has been incorporated. |
| 9 | The table is incomplete. The variables also need to mention the number of children in each category. Heading ....two more column need to be added in table...One for case (n) another for control (n). | Changes have been incorporated, as suggested. Two columns have been added in table 3, as suggested by you. |
| 10 | Is it during initial 6 months after delivery | Thank you for the comment. This is within 45 days after delivery as per the protocol of Ministry of Health and Population. Timeframe has been updated on Table 1: Categories of Variables under study. |
| 11 | How many children are there ....with care givers other than mother | Out of the total children, 9% were looked after other mothers. Values have been updated in Table 3 as suggested. |
| 12 | Duration which period ? | This is within one month of survey. Timeframe for occurrence of diarrhea has been updated on Table 1: Categories of Variables under study. |
| 13 | "strong study design" is a vague word it needs to deleted | Deleted as suggested. We have replaced the phrase ‘strong study design’ with ‘robust study design’ in the ‘study limitations’ sub-section of the revised manuscript. |
